# Adolescents Searching for Health Information on the Internet: An Observational Study

**DOI:** 10.2196/jmir.5.4.e25

**Published:** 2003-10-17

**Authors:** Derek L Hansen, Holly A Derry, Paul J Resnick, Caroline R Richardson

**Affiliations:** ^1^School of InformationUniversity of MichiganAnn Arbor MIUSA; ^2^Health Media Research LaboratoryUniversity of Michigan Comprehensive Cancer CenterAnn Arbor MIUSA; ^3^Department of Family MedicineUniversity of Michigan Medical SchoolHealth Services Research and DevelopmentVA Medical CenterAnn Arbor MIUSA

**Keywords:** Internet, adolescent, access to information, medical informatics, computer simulation, computer literacy, health education, search process, information seeking, information storage and retrieval

## Abstract

**Background:**

Adolescents' access to health information on the Internet is partly a function of their ability to search for and find answers to their health-related questions. Adolescents may have unique health and computer literacy needs. Although many surveys, interviews, and focus groups have been utilized to understand the information-seeking and information-retrieval behavior of adolescents looking for health information online, we were unable to locate observations of individual adolescents that have been conducted in this context.

**Objective:**

This study was designed to understand how adolescents search for health information using the Internet and what implications this may have on access to health information.

**Methods:**

A convenience sample of 12 students (age 12-17 years) from 1 middle school and 2 high schools in southeast Michigan were provided with 6 health-related questions and asked to look for answers using the Internet. Researchers recorded 68 specific searches using software that captured screen images as well as synchronized audio recordings. Recordings were reviewed later and specific search techniques and strategies were coded. A qualitative review of the verbal communication was also performed.

**Results:**

Out of 68 observed searches, 47 (69%) were successful in that the adolescent found a correct and useful answer to the health question. The majority of sites that students attempted to access were retrieved directly from search engine results (77%) or a search engine's recommended links (10%); only a small percentage were directly accessed (5%) or linked from another site (7%). The majority (83%) of followed links from search engine results came from the first 9 results. Incorrect spelling (30 of 132 search terms), number of pages visited within a site (ranging from 1-15), and overall search strategy (eg, using a search engine versus directly accessing a site), were each important determinants of success. Qualitative analysis revealed that participants used a trial-and-error approach to formulate search strings, scanned pages randomly instead of systematically, and did not consider the source of the content when searching for health information.

**Conclusions:**

This study provides a useful snapshot of current adolescent searching patterns. The results have implications for constructing realistic simulations of adolescent search behavior, improving distribution and usefulness of Web sites with health information relevant to adolescents, and enhancing educators' knowledge of what specific pitfalls students are likely to encounter.

## Introduction

The Internet has become an important tool for many people with health concerns [1,2], especially for adolescents [3,4]. Concerns about confidentiality, coupled with the fact that many teenagers find accessing care through traditional providers difficult [5], make access to information via the Internet particularly important. Given rapidly-expanding Internet access among young people, it is not surprising, then, that more than 70% of 15 to 17 year-olds say they have used the Internet to look up health information (written communication, 2001 Dec; Generation RX.com Survey printouts; V. Rideout, Henry J. Kaiser Foundation, Menlo Park, CA). This percentage is likely to increase if Internet access from home continues to rise as it has in recent years [6].

Because of the enormous amount of unstructured online content, it is crucial to understand how youth navigate through the Web to find health information. Prior research, primarily from library and information science literature and education literature, has highlighted several search characteristics that are either unique or more pronounced in adolescents. For example, adolescents take more time to complete online tasks than college students [7], search less systematically [7- 10], have difficulty formulating search queries due to misspelling and problems with the level of specificity [8- 11], utilize less-advanced search syntax [7], and rarely consider the source of Web pages [8,9]. While informative, this literature is based primarily on adolescents searching for answers to homework questions rather than health information.

Searching for online health information involves distinctive challenges including unfamiliar terminology [12]; encounters with pornography-blocking software (written communication, 2001 Dec; Generation RX.com Survey printouts; V. Rideout, Henry J. Kaiser Foundation, Menlo Park, CA), [13]; and the importance and difficulty of determining health information quality [14]. However, despite the need for research that details the online search behavior of health consumers, the authors were only able to locate a few articles in which health science researchers actually observed, recorded, and analyzed consumers of any age searching for health information [14- 16]. Instead, surveys (eg, written communication, 2001 Dec; Generation RX.com Survey printouts; V. Rideout, Henry J. Kaiser Foundation, Menlo Park, CA; and [1,2,4]) have been the predominant method used to understand health consumers' online searching behavior, despite problems with participant recall and the inability of surveys to capture specific search tactics. In addition, the authors found a handful of studies in the medical informatics literature that have also looked at log data from particular medical Web sites, but these studies are also limited in scope since they do not observe the actual searcher or see the broader context in which the searcher is acting [17] (see also [18] for similar studies performed on search engine data). The value of directly observing users was demonstrated in the Eysenbach study, which revealed that adults said they paid attention to the source of health sites during interviews, although this behavior was not found during the actual observations [15].

Observational research specific to the adolescent age group and online search behavior for health information is also sparse. There have been some good surveys that answer many useful questions concerning why adolescents go to the Internet, what they search for, if they find it, and what they do with it (written communication, 2001 Dec; Generation RX.com Survey printouts; V. Rideout, Henry J. Kaiser Foundation, Menlo Park, CA;and [4,19]). The only observational study we were able to locate conducted 27 focus groups where groups of adolescents searched online for health information as they discussed their own experiences [14]. Many of the findings concerning adolescent search behavior found in the library and information science literature were confirmed and additional issues were raised, including concerns about low health literacy and trouble judging the quality of information, that may be more pronounced in adolescents than adults. However, that study only begins to paint a picture of adolescent search behavior for online health information, because the searches were performed in a group setting and the success, failure, and specific search tactics used were not coded or analyzed.

The study reported here provides a more in-depth understanding of how adolescents search for health information using the Internet and what implications this may have on access to health information. To capture enough detail, the study recorded specific actions taken by adolescents which were later coded and analyzed. Participants were encouraged to share their thought process out loud as they searched for answers to a list of predetermined health questions. The result was a rich set of both quantitative and qualitative data that was thoroughly analyzed for common themes and events. Specific questions of interest include, but are not limited to: What are the various search strategies used? What factors contribute to finding correct and useful answers? When using a search engine, how many results pages are viewed and utilized? What types of search strings are entered into search engines? Answers to these and related questions should be of interest to a number of parties including educators (eg, health educators, librarians, teachers), Web site and search engine designers, health care practitioners, and researchers (eg, to create a sample of URLs by simulating online searching behavior [20]).

## Methods

### Sample

Twelve students from 1 middle school (N= 4) and 2 high schools (N = 4 and N= 4) in southeast Michigan were recruited for this study. Staff at each school were asked to select 4 students who were (a) comfortable using computers, (b) comfortable searching for information on the Internet, and (c) strong students who could afford to miss one class period. Students received a University of Michigan T-shirt, valued at roughly $8, in return for their participation.

The parent or guardian of every student signed an informed consent document that described the purpose and procedure of the study. Students also signed separate assent forms with similar information. The University of Michigan Behavioral Science Institutional Review Board approved this study and the consent and assent documents.

### Data Collection

Three methods of data collection were used. First, one of the two members of the research team present during each of the observations coded searching behavior in real time while the second member of the research team interacted with the student. Second, TechSmith Camtasia 3.0.1 commercial tracking software [21] was installed on the computer. This software captured the students' voices and took pictures of the screen (screen captures) twice per second during the entire session. Finally, a video camera was positioned to capture the screen and the students' voices, but not the students' images. Observations coded in real time were used to develop a more detailed and systematic coding system for use when reviewing the tracking software records. It is data from the tracking software coding that is reported here.

All observations of adolescents were conducted during January 2002. Each school provided a room in which to conduct the observations. Students were brought to the observation room one at a time. Two researchers were present at every observation. For each student, one of the researchers first reviewed the assent form to introduce the project and obtain the student's permission to participate. The students were then asked 14 questions about demographics (age, race/ethnicity, and gender) and their prior computer use (eg, how often they use computers or the Internet, what health topics they have searched, which search engines they used, and whether they have a computer and access to the Internet at home).

Once the brief interview had been completed, the observed searches began. To help the students understand the procedure and to reinforce the importance of thinking out loud while doing their searches, each student was first asked to do an easy non-health-related search looking for the next day's local weather forecast. As with the subsequent health-related searches, the local-weather question was first read to the student by a researcher and then a card with the question on it was set next to the computer in case the student needed to read it. As part of the think-aloud protocol, the experimenter asked the student to talk out loud about what they were doing, so that researchers could better understand the reasons behind the searching behavior. If a student stopped talking during the search, he or she was reminded by the observers to "keep talking," but the experimenters did not ask students to elaborate on any specific thing they said. Concurrent verbal reports more accurately reflect a subject's mental state at the time of observed behaviors than do retrospective reflections, and this minimal think-aloud protocol has been shown to slow subjects down, but not to qualitatively change their problem solving behavior [22].

After the students completed the practice local-weather search, they were given a sequence of up to 6 predetermined health information questions (see Table 1), 1 at a time. Questions were framed in a way that took into consideration the broader information concern that the question attempted to resolve. To eliminate confounding by learning effects between searches, we used a 6 x 6 Latin square to determine the order in which the questions were presented to the participants. The computer that students used was provided by the researchers, but connected to the school's network so that the students were protected from controversial or pornographic material by the same blocking or filtering software used by the school. The 3 different schools used 3 different filtering systems. Each observation session lasted one class period. No time limit was given for each question, but when the class period ended, any ongoing search was terminated and any remaining questions were skipped.

**Table 1:** Health-related questions

Your aunt was just told she has diabetes. She isn't sure what kinds of food she can or can't eat. Using the Internet, find some information for your aunt about what foods she should or should not eat.
A friend recently started taking a drug called Paxil for depression. He seems to be tired all the time, and even falls asleep in class. Use the Internet to find out if the drug might be making him sleepy.
Your older brother has a problem with drinking too much alcohol. He wants to go to a local Alcoholics Anonymous meeting. Use the Internet to help him find a local meeting.
You want to get an HIV test, but you don't want anyone to know. You also don't have any money to pay for it. Use the Internet to find a place to get a free and confidential HIV test.
For class, you need to learn about medicine that can help people stop smoking. Using the Internet, find the names of these medicines.
You are about to get a tattoo, but a friend warned you that some places spread infections like HIV and hepatitis. Use the Internet to find out if this is true.

Topics for the health-related questions were chosen based upon responses to a survey of adolescents conducted by the Kaiser Family Foundation (written communication, 2001 Dec; Generation RX.com Survey printouts; V. Rideout, Henry J. Kaiser Foundation, Menlo Park, CA). Certain topics including homosexuality, teen pregnancy, and abortion were purposefully avoided so as not to expose participants to overly-controversial information.

### Data Analysis

After all the observations were completed, 3 researchers including a physician, health educator, and human-computer interface specialist met as a group to review the real-time coding results and to clarify or augment the coding scheme before the definitive final coding of the tracking-software records. The final coding scheme was designed to record data on the person searching, the question being asked, the time it took to find an answer, the search strategy utilized (eg, utilize search engine or directly type in URL); search strings used; number of search engine results pages reviewed; number of pages viewed within a particular site; and the use of menus, advertisements, and directories. One of the 3 coders was assigned as a primary reviewer for each of the observation sessions. The assigned primary reviewer was responsible for a detailed coding of the observation session and any coding problems were resolved in a second group discussion.

The reviewers classified each of the answers found by the students as *correct* or *incorrect*, *complete* or *incomplete*, and, for location questions only, *useful* or *not* *useful.* To avoid being overly narrow in our classification of *correct* for the more open-ended questions such as the question on healthy foods for a person with diabetes, we used the following general rule for classification: to be considered correct, the content of the answer had to be the kind of information that might be discussed in a medical school or school of public health. This classification system was validated in previously-published work by the research team and resulted in a high inter-rater reliability (κ = 0.84) [13]. The more-specific questions such as the question asking about a location for an Alcoholics Anonymous meeting were considered correct if the student found a Web page listing a meeting location and time or contact phone number. Answers were complete if the students were able to answer all parts of the question. For example, if the student found a discussion about HIV transmission by tattoo parlors, but did not find an answer about hepatitis it was classified as incomplete. Useful answers pertained to location questions. An Alcoholics Anonymous meeting in another state was not useful. A summary measure classifying each search as successful, partially successful, or unsuccessful was computed using the correct, complete, and useful ratings. To obtain a rating of successful, the answer had to be complete, correct, and useful. If the student gave up before finding an answer, the search was classified as unsuccessful.

## Results

Twelve middle school students and high school students in southeast Michigan participated. Students ranged in age from 12 to 17 years old, with a mean of 14 years. Half of the students were female. Of the 12 students, 7 were white, 2 were African American, 1 was Indian American, 1 was Hispanic, and 1 was Asian American. Of the 12 students, only the 6 oldest students had searched for health information on the Internet before. The variation by age is consistent with other findings that youth age 15 to 17 years are significantly more likely to have looked up health information (32%) than youth age 12 to 14 years (18%) [23]. All of the students, however, had computers and access to the Internet at home. Students reported using a computer from 1 hour per week to 3 hours per day, with a mean of 12.3 hours per week.

Eleven students attempted all 6 searches, while the remaining student attempted 3, for a total of 69 searches. One search was not included since the Internet connection was not working properly, making a total of 68 searches that were analyzed. Searches took an average of 5 minutes and 41 seconds, ranging from just under a minute to nearly 24 minutes. This time frame is essentially the same as Eysenbach recorded for adults [15]. Although direct comparison is inappropriate since different questions were asked, the similar order of magnitude is suggestive.

### Overall Search Strategy

As students thought aloud, the researchers got a sense of what students were looking at on each page. Students seemed to skip around a lot, and didn't skim results pages or specific Web sites in any methodical or thorough ways, sometimes missing links or text that contained the answer to questions. This is also consistent with findings from non-health-related searching behavior as summarized in Hsieh-Yee [24].

**Table 2:** Distribution of pages viewed per site

**Pages Viewed Per Site**	**Sites**		
	**n**	**%**	**Cumulative %**
1	143	70.4	70.4
2	27	13.3	83.7
3	11	5.4	89.2
4	8	3.9	93.1
5	8	3.9	97.0
6	2	1.0	98.0
8	1	0.5	98.5
9	1	0.5	99.0
15	2	1.0	100.0
Total	203	100	

Students used multiple methods to locate Web sites that they believed contained answers to the 68 questions. In 60 cases, the student started looking for an answer by visiting a search engine and entering in a search term or phrase. In 2 cases, the student started by selecting from directory menus (eg, choosing the topic *health*). In 6 cases, the student started by entering a URL (other than a search engine) directly into the browser address bar. In total, there were 215 attempts to access non-search-engine or directory Web sites. Nearly all of these attempts were made by following a link from a search engine either after a search or through the use of a directory. Of the 215 attempted site visits, 4 were broken links, 3 were blocked by the filters utilized at certain schools, and 5 were PDF files (read by Acrobat Reader) which students either could not download or chose not to download because downloading was too slow. This left 203 sites that were viewed with an average of 1.8 pages viewed per site. The distribution of pages visited per site is shown in Table 2. Note that the distribution is roughly consistent with a power law as observed in previous studies [25]. At a reviewer's request, this data was looked at on an individual student level. Students varied a great deal in the total number of visited sites. Eleven of the 12 students went only 1 page deep on the majority of visited sites. Although the individual-level data is not large enough to analyze more rigorously, the power law seems to operate on an individual level as well as the aggregate level.

Even when students found a Web site that contained the answer to a question, they did not always find the answer. One example is the Alcoholics Anonymous site [26] where 8 of the 11 students ended up while searching for a local meeting. Although there was a link to a site that contained local information, only 3 of the 8 students were able to find the link, 1 of whom only found it on the second visit to the Alcoholics Anonymous site, after viewing a total of 16 pages within the site. Similarly, 6 of the 11 students who searched for whether or not Paxil causes drowsiness visited the official Paxil site [27]. Only 3 of the 6 students were able to successfully answer the question based upon the information they found at the site. Two of them failed to find the list of side effects and 1 of them found the list but did not understand it enough (or read it carefully enough) to answer the question correctly.

### Search Engine Tactics

Seven search engines were used, including 2 meta-search engines (Dogpile and Locate.com). The meta-search engine Locate.com offers the user a number of search engines to choose from. Searches performed from the Locate.com Web site that utilized another search engine (eg, Yahoo!) are reported as if the search occurred on the destination search engine (eg, Yahoo!). Table 3 summarizes the number of times that a particular search engine was used. If a search engine was used multiple times while searching for an answer to the same question, it is only counted once. Because students occasionally switched search engines while trying to answer the same question, there are more searches using a search engine (79) than there are attempts to answer questions (68). In total, 6 of the 12 students used only Google, 1 used only Yahoo!, and the remaining 5 changed search engines at some point.

**Table 3:** Search engine usage

**Search Engine**	**Times Used**	
	**n**	**%**
Google	38	48.1
Yahoo!	13	16.5
Ask	12	15.2
MSN	7	8.9
Hotbot	6	7.6
Dogpile	2	2.5
AltaVista	1	1.3

A total of 132 search phrases were entered into the various search engines. Only 104 of those search phrases were unique. The most-frequent 2 phrases used were "diabetes" and "Paxil," each of which had 5 occurrences. There was an average of 3.6 words typed in per search phrase and 80% of the time there were 4 or fewer words per search phrase.

**Table 4:** Distribution of search-result links viewed

**Bands of Search-Result Links Viewed**	**Chosen Links**		
	**n**	**%**	**Cumulative %**
Results 1-10	137	82.5	82.5
Results 11-20	8	4.8	87.3
Results 21-30	11	6.6	94.0
Results 31-40	4	2.4	96.4
Results 41-50	4	2.4	98.8
Results 51-60	1	0.6	99.4
Results 61 or more	1	0.6	100.0

Of the 132 search phrases, 30 contained at least 1 word that was misspelled (eg, "tatoo," "Alchoholics," or "smokeing"), despite the fact that students could read the correctly-spelled word on the index card containing the question. Some search engines (eg, Google) offer a feature that recommends an alternate search string with the correct spelling of a word. For example, if a student typed "alchoholics anonymous," the first page of results began with, "Do you mean 'alcoholics anonymous?'" Students were offered a new search string with correct spelling on 15 separate occasions, but only noticed and used it 6 times. The remainder of the times they used the results that were offered for the incorrect spelling. Of the 7 students who were offered corrected spelling suggestions, only 2 ever used them.

Once a search string was entered into a search engine, students varied in the number of results pages that were viewed. Students viewed only the first results page 78% of the time and 4 pages or less of results 93% of the time. Because search engines report a different number of links per page of search results, Table 4 reports how often links were selected from the first 10 results, the second 10, and so on. Only 3 blocked links were encountered during all of the searches, suggesting that blocking software did not have a significant impact on these results.

### Successful Searching Characteristics

Of the 68 questions that students attempted to answer, 7 searches were abandoned after the student gave up or, in 2 cases, when the class period ended. Of the remaining 61 searches, 47 were successful in finding a complete, correct, and useful answer to the health question and the remaining 14 were unsuccessful. Six of the unsuccessful answers were completely incorrect and not useful, 4 were useful but only partially correct, and 4 were fully correct but not useful.

Several factors contributed to the success of finding a correct, complete, and useful answer. One important factor was the individual who was performing the search. Although every student answered at least 1 question correctly there was wide variation in the number of correct answers. Two students successfully answered 6 out of 6 questions, 3 students successfully answered 5 questions, 4 students successfully answered 4 questions, and the remaining 3 students only successfully answered 1 or 2 questions. While our sample of students was too small to draw conclusions from, no distinct patterns were observed that would indicate that race, gender, Internet experience, or health searching experience were significant determinants of success. However, the older adolescents (16-17 year olds) were successful 87% of the time (26 of 30) as compared to 68% (21 of 31) for the younger adolescents.

Another important factor was the difficulty level of the questions themselves. Table 5 shows the failure rate for each question. The 4 partially-correct answers were split evenly between the Alcoholics Anonymous and tattoo questions. All 4 of the correct but not useful answers resulted from the HIV test question.

**Table 5:** Unsuccessful searches by search topic

**Search Topic**	**Unsuccessful Searches**	
	**n**	**%**
HIV test	8	38.1
Paxil	4	19.0
Alcoholics Anonymous	3	14.3
tattoo	3	14.3
smoking	2	9.5
diabetes	1	4.8
Total	21	100.0

Certain search actions led to sites that contained the answer more often than others. Overall, students found answers on 22% of the sites they accessed (47 of 215). They accessed sites in 5 ways. Although not often taken, the action with the highest probability of success (47%; 7 of 15) was following a link from 1 non-search-engine site (eg, www.aa-intergroup.org) to another site (eg, www.alcoholics-anonymous.org). In most of these cases, the student accessed the first site directly from a search engine. Clicking on search engine results led to a site where students found an answer 21% of the time (35 of 166). Success rates were similar for following a recommended link from a list or menu provided by the search engine (18%; 4 of 22). Directly typing in a URL, bypassing search engines entirely, was successful only 9% of the time (1 of 11). A sponsored link from a search engine was followed only once, and the student found an incorrect answer on that site.

Another contributing factor related to success was misspelling of search terms. Of the 14 completed but unsuccessful searches, 29% (4 searches) had at least 1 misspelling compared to only 15% (7 searches) of the 47 successful searches. Perhaps even more telling, both successful and unsuccessful searches with misspellings took students 1.5 minutes longer on average than searches without misspellings. Observations confirmed that some students were unable to find an answer until they discovered and corrected their misspelling, resulting in higher quality and more-relevant results.

Other search characteristics did not have statistically significant impacts on whether searches were successful, although this may have been due to small sample sizes. For example, the search engines were not significantly different in their percentages of successful searches. Similarly, the average number of words per search string was not significantly related to search success rate. (Data not shown.)

### Qualitative Analysis

Certain common behaviors of the adolescent searchers were observed which were not apparent from the quantitative analysis.

First, the students were very comfortable and confident while searching online for health information. Most students knew where they wanted to start the search and navigated using quick mouse clicks and shortcut keys. However, this characteristic was likely over-represented in our population due to their strong academic performance and Internet proficiency.

Second, several searchers did not take much time in formulating a search strategy or (when applicable) choosing search terms. Instead, these searchers seemed to type in the first search string that came to mind. If the results were not what were anticipated, another search string was typed in, sometimes without even clicking on any results from the first search string. The overall approach was a trial-and-error method with frequent backtracking. The most-common problem with search strings was that they were not specific enough. For example, 2 different students typed in the search string "hiv" when looking for a place that administers free and confidential HIV tests.

Third, most students quickly scanned pages, jumping from place to place within a page, rarely reading an entire paragraph. In some cases the answer to a question was contained on a page, but the student left before finding it. In other cases a link that would have led to the answer was missed. This finding supports prior research on adolescent search behavior related to nonhealth topics [7- 10].

Fourth, students mentioned that they purposefully avoided sponsored links and advertisements, despite the fact that many of the search engines present these results first. The qualitative data confirmed this practice, as only 1 sponsored link was ever selected.

Finally, little to no attention was paid to the source of the answer. In the vast majority of cases, once an answer was located, it was simply assumed to be correct.

## Discussion

When compared with prior research, the findings of this study show many similarities and a few key differences between the behaviors of adolescents and adults while searching for health information. This study found that adolescents searching for health information utilized search engines nearly every time. This finding was similar to that for adults as described in the Eysenbach study [15]. These observational studies also suggest that after-the-fact survey questions concerning the use of search engines may underestimate this behavior. For example, 2 nationally-representative surveys reported that 58% of youth (written communication, 2001 Dec; Generation RX.com Survey printouts; V. Rideout, Henry J. Kaiser Foundation, Menlo Park, CA) and 81% of adults [1,2] started seeking health information at search engines. Our study found that adolescents relied upon links from only the first few results pages, and rarely explored far within any site. These results also were similar to adult searching behaviors [15], although youth seem to be more likely to search beyond the first 10 search results. Adolescents often chose search strings that were too general and/or contained misspellings, so that they did not always find useful sites that were available. Eysenbach also reported search strings by adults that were too general [15], however, spelling seems to be more of a problem with youth. Adolescents were unsystematic in their reading of Web sites and some sites were poorly organized so that they did not always find the information they were looking for, even when it was present in a site they examined. Future research is needed to better understand if adolescents do not understand information provided on these sites, whether they simply have less patience, or some other explanation. In summary, many of the specific search tactics are similar for adults and adolescents, but a few issues related to spelling, browsing of Web sites, and understanding of content are notably different.

### Simulation of Searches

The results from this study have implications for anyone who simulates adolescent health searches, for providers of health information, and for educators. There are many reasons to simulate adolescent health searches. For example, an educator preparing a lesson plan may want to informally simulate searches in order to anticipate what students are likely to find if given certain particular search tasks. A researcher may want to simulate adolescent searches more systematically to evaluate the availability and accessibility of information on particular topics, to evaluate which search engines should be recommended to adolescents, or to evaluate whether the installation of filtering software will have a detrimental impact on accessibility of health information [13]. Because many of the search behaviors modeled by these simulations are similar for both adolescents and adults, results from studies that simulated one or the other group likely apply to both groups.

The results of this study suggest that such simulations can focus on the use of search engines, but that very-broad search terms and, especially for adolescents, common spelling errors should be considered. Ads and other nonresult links can be ignored. Since more than 80% of the links that were followed appeared in the top 10 results, and more than 95% were among the top 40, a search simulation need not consider result links beyond these.

### Providers of Internet Health Content

Given the patterns of adolescent searching behavior found in this study, providers of health content can do several things to increase the probability that adolescents will find their sites. Since adolescents rely primarily on the first few results from search engines and do not tend to look at ads, it is important to ensure that health sites appear near the top of the results for searches on health terms. Choices of keywords in the domain name, page title, meta tags, and the first few sentences, as well as links from other sites, can all affect placement in search results. It may also be useful to include some common misspellings in meta-tag keywords and in the body of the text in order to make a site appear in the results page of searches using those misspellings of related search terms. Because most major English-language search engines no longer use the keyword feature of meta tags, site designers are left with the difficult task of working misspelled words (eg, misspelt) into the text without coming across as poor spellers themselves. It is also important that the site descriptions displayed in search engines be attractive to adolescent searchers: while our study did not analyze the various reasons that adolescents chose to follow one link over another, we did observe that they made choices based upon the link descriptions and did not simply select the first link offered. Books and articles, software, and consulting services are all widely available to improve search engine placement and to influence the short summary text that search engines extract for display in search results [28,29]. Organizations that invest large amounts of money in developing sophisticated health-information sites would do well to spend a little bit more to ensure these sites are easily found.

Another area that Internet content providers should focus on is within-site navigation. Because students tend to skip around from place to place within a page and read little in sequence, it is important that sites with a significant adolescent audience are well organized, concise, and understandable. Long paragraphs, too many links, and difficult vocabulary all decrease the likelihood of adolescents finding health information they are seeking, even if it is contained within a site. Internet content producers should attempt to understand the needs of the site visitors and build hierarchal structures that reflect those needs. For example, if one of the primary needs of individuals visiting the Alcoholics Anonymous site is to find a local meeting, the first page of the site should include an obvious link (eg, "Find an AA Meeting Near You") that leads to another page that returns the nearest meetings after entering in a zip code or city name. While ease of within-site navigation is important for all visitors to health information sites, some information providers may want to develop sites targeted specifically to adolescents. While they might like the targeted information once they found it, we observed that adolescents tend to rely on general-purpose search engines. Thus, developing special youth-targeted versions of information sites may be of somewhat limited utility, unless also accompanied by advertising or education campaigns that make adolescents more likely to find such sites.

Rather than changing Web sites or their presentation in search engines, it may also be useful to undertake education campaigns to improve the search strategies and tactics that adolescents use when seeking health information. It may be helpful to guide them towards youth-oriented directories or search engines, rather than general-purpose search engines. For example, both Yahoo! and Google offer directories with subcategories of sites designed for teens that cover various health topics. This approach may be facilitated by including links to such resources on the Web browser's starting page in schools and libraries. Alternatively, adolescents might be taught techniques for formulating and refining search terms at general-purpose search engines, adding or dropping more-specific words based on the kinds of results returned. They might also be taught to notice potential search term misspellings based on surprising search results. Finally, adolescents might also be taught techniques for systematically exploring within a Web site to find the kind of information they are looking for.

### Limitations and Future Research

There are several important limitations to the interpretation of these results.

First, this was not a representative or random sample of adolescents. It was a small convenience sample with a selection bias toward adolescents with strong Internet searching skills. While the results cannot be generalized to all adolescents and do not capture the full range of adolescent searching experience, we can assume that the average adolescent would have had even more trouble than our study participants in finding health information on the Internet.

Second, the health-related search questions were deliberately constructed to avoid controversial topics such as safe sex, abortion, and homosexuality. Given that adolescents are often faced with health problems related to sexuality, their actual search behavior and success at finding health information related to sexuality may not be reflected in our results. Another concern is that participants may have changed their search behavior because of the presence of observers and because they were aware that their search behaviors were being recorded. For example, students who had trouble finding an answer may have persisted in their search longer than they would have in a nonresearch setting. Alternatively, because students knew they had several search questions to answer during a single class period, they may not have been as persistent as they might have been with a more personally-relevant question and less-restricted search time. Thus, the data here reflect a rough estimate of persistence for an adolescent looking for health-related information. Also, searching was conducted individually, while in practice many searches both at home and at school are conducted with friends, teachers, or family close by. While it is difficult to know how this would affect searching behavior without future research, it is possible that students would act differently (eg, receive help with spelling).

Finally, while components of our classification scheme for successful versus unsuccessful searching have been previously validated, the overall scheme was modified to more accurately code the search results as correct, complete, and useful. A more-systematic validation of coding schemes for health information search results is an important area for future research.

More research is needed to validate the results presented in this article, as well as determine if results vary for different populations (eg, age, race, and experience with health searching) and different health questions (eg, finding a practitioner versus finding the answer to a question). Additionally, instead of focusing on how adolescents currently search for health information, future studies may also want to explore interventions aimed at improving their searches. For example, should health portal sites designed for adolescents or online directories be used? Or would the current practice of using common search engines, but with adolescents learning improved search tactics be more effective? Also, which search strategies lead to sites that are the most likely to be accurate and influence adolescents to change their behavior?

### Conclusions

This study provides a useful snapshot of current adolescent searching patterns. The results have implications for constructing realistic simulations of search behavior, and for both information providers and educators. Analyzing search behavior through actual observation should be a cornerstone in any effort to improve adolescents' access to health information.
